# Proteomic analysis of stage I primary lung adenocarcinoma aimed at individualisation of postoperative therapy

**DOI:** 10.1038/sj.bjc.6604197

**Published:** 2008-01-22

**Authors:** J Maeda, T Hirano, A Ogiwara, S Akimoto, T Kawakami, Y Fukui, T Oka, Y Gong, R Guo, H Inada, K Nawa, M Kojika, Y Suga, T Ohira, K Mukai, H Kato

**Affiliations:** 1 Department of Surgery, Tokyo Medical University, 6-7-1 Nishi-shinjuku, Shinjuku-ku, Tokyo 160-0023, Japan; 2 Clinical Proteome Center, Tokyo Medical University, 2-6-1 Nishi-shinjuku, Shinjuku-ku, Tokyo 163-0217, Japan; 3 Medical ProteoScope Co. Ltd, 2-6-1 Nishi-shinjuku, Shinjuku-ku, Tokyo 163-0217, Japan; 4 Taiho Pharmaceutical Co. Ltd, Tokyo, Japan; 5 Department of Pathology, Tokyo Medical University, 6-7-1 Nishi-shinjuku, Shinjuku-ku, Tokyo 160-0023, Japan

**Keywords:** myosin IIA, vimentin, postoperative adjuvant chemotherapy, responder to uracil–tegafur, stage I lung adenocarcinoma

## Abstract

Although postoperative adjuvant chemotherapy (PAC) with uracil–tegafur significantly improves the prognosis of patients with stage I lung adenocarcinoma, subset analysis has revealed that only 11.5% of patients with stage IB derive actual benefit from such therapy. Therefore, it is extremely important to identify patients for whom adjuvant chemotherapy will be beneficial. We performed comprehensive protein analysis of 24 surgically resected specimens of stage I adenocarcinoma using liquid chromatography-tandem mass spectrometry (LC-MS/MS), followed by bioinformatical investigations to identify protein molecules. Furthermore, we carried out immunohistochemical studies of 90 adenocarcinoma specimens to validate the results of LC-MS/MS. We detected two kinds of protein molecules (myosin IIA and vimentin) by LC-MS/MS. We confirmed their immunohistochemical expression and distribution, and evaluated the relationship between the expression of these proteins and prognosis after adjuvant chemotherapy. Patients with no expression of either myosin IIA or vimentin showed a significantly better outcome regardless of PAC using uracil–tegafur. However, we were unable to select responders to uracil–tegafur using these proteins. Cases of adenocarcinoma lacking expression of either myosin IIA or vimentin show a good outcome without PAC, and therefore do not require such treatment.

Death due to lung cancer is still increasing in most industrialised countries, including Japan, despite improvement of various diagnostic and therapeutic modalities. Even though the opportunities to detect lung cancer at an early stage are increasing, approximately 60 000 patients with lung cancer die every year in Japan, usually due to distant metastasis. Distant metastasis, including intrapulmonary metastasis, frequently occurs in patients with advanced-stage non-small cell lung cancer (NSCLC) who undergo only surgical resection, because in such cases micrometastases probably exist at the time of surgery. The concept of postoperative adjuvant chemotherapy (PAC) for control of micrometastasis does not conflict with the improved prognosis of NSCLC patients. However, the efficacy of PAC in patients after complete resection of NSCLC was a matter of controversy in the 1990s. Even as recently as 2003, the efficacy of PAC could not be demonstrated (Scagliotti *et al*, 2003). In 2004, however, some studies demonstrate a beneficial effect of PAC (Arriagada *et al*, 2004; Strauss *et al*, 2004; Winton *et al*, 2005). We have also reported that PAC with oral uracil–tegafur (DPD Inhibitory Fluoropyrimidine, Taiho Pharmaceutical Co. Ltd, Tokyo, Japan) provided better survival than surgical treatment alone in patients with stage I adenocarcinoma of the lung (Kato *et al*, 2004). The combination of uracil and tegafur (also referred to as UFT) at a molar ratio of 4 : 1 is an oral anticancer agent with good absorption in the small intestine (Fujii *et al*, 1979). Tegafur is a prodrug that is gradually converted into fluorouracil in the liver by the cytochrome P-450 enzyme system. Uracil enhances the serum concentration of fluorouracil by competitive inhibition of dihydropyrimidine dehydrogenase, the enzyme responsible for fluorouracil catabolism (Ikenaka *et al*, 1979). Oral uracil–tegafur generates a higher maximal plasma level of fluorouracil than protracted intravenous infusion of fluorouracil at a dose that is equimolar to the amount of tegafur in uracil–tegafur (Ho *et al*, 1998).

Even though PAC with uracil–tegafur has significantly improved the prognosis of patients with stage I primary lung adenocarcinoma, even subset analysis of stage IB has revealed that 11.5% of patients actually derive some benefit from the treatment (Kato *et al*, 2004). Nonresponders to uracil–tegafur, including relapse-free patients without any adjuvant therapy, gain no benefit from PAC. In this context, it is important to establish biomarkers for prediction of responders to uracil–tegafur, and/or for favourable prognosis without the use of PAC.

Clarification of the entire human genome is one of the most significant events in the history of bioscience, and has accelerated the comprehensive analysis of human genes and their protein products. Many biologists recognise the importance of protein analysis, because proteins play central role various cellular functions. However, in cancer research, there has been a tendency for most researchers to avoid investigation of cancer-related proteins, because their structures are more complicated than those of the genes. Nevertheless, techniques for the comprehensive analysis of proteins have improved greatly in recent years. The concept of comprehensive protein analysis has been established, and the new research field of proteomics has been developed. One of the main purposes of clinical proteomics in the field of oncology is the development of new therapeutic strategies for cancer, centred on individualised therapy. In this study, we attempted to identify biomarkers for the selection of responders to uracil–tegafur and nonresponders, including relapse-free patients without any requirement for PAC, using clinical proteomics methodology.

## MATERIALS AND METHODS

### Institutional review board approval for this investigation

The institutional review board approved the use of proteomics analysis to explore biomarkers for selection of responders to oral uracil–tegafur (294/323/480/702).

### Surgical samples of stage I lung adenocarcinoma for mass spectrometry

After obtaining written informed consent, lung cancer tissues were obtained from patients with pathologically confirmed stage I adenocarcinoma resected at Tokyo Medical University Hospital between 1995 and 2001. Tissues were kept frozen at −80°C until use. We collected 11 lung adenocarcinoma specimens from 11 patients who subsequently underwent PAC using uracil–tegafur for more than 2 years. In 5 of these 11 patients, recurrent lesions were detected within 2 years after surgery (U1R1), and the remaining 6 were confirmed to be disease-free for 5 years after surgery (U1R0). Furthermore, 13 specimens of lung cancer were collected from patients receiving no adjuvant therapy after surgery. In 6 of these 13 patients, recurrence was recognised within 2 years after surgery (U0R1), but no recurrent lesions were detected during 5 years after surgery in the other 7 (U0R0).

### Protein extraction

The surgically resected materials were suspended and homogenised in PBS supplemented with a protease inhibitor cocktail (Roche Diagnostics Inc., Basel, Switzerland) at 4°C. The cell lysate was then fractionated by ultracentrifugation (52 000 **g**, 4°C, 20 min). The resulting pellet containing plasma membranes from the cells, was solubilised in PBS containing 5% SDS with continuous ultrasonication. The resulting solution was taken as the insoluble fraction, whereas the supernatant from the ultracentrifugation, containing mainly cytosolic proteins, was taken as the soluble fraction. The total protein concentrations of both fractions were measured (Lowry *et al*, 1951) using bovine serum albumin as a standard.

### Protein condensation with SDS-PAGE

We added 150 pmol egg white lysozyme (Sigma-Aldrich Inc., St Louis, MO, USA) to an aliquot containing 75 *μ*g protein from each fraction, then dried it under vacuum. The mixture was then solubilised in sample buffer (Laemmli, 1970) with gentle stirring at 37°C for 1 h. A two-third volume of the solution containing 50 *μ*g sample protein and 100 pmol lysozyme was subjected to SDS-PAGE on 12.5% polyacrylamide gel 1-mm-thick. SDS-PAGE was carried out at a constant current of 20 A until the bromophenol blue marker passed the boundary between the stacking and separation gels. In this ‘halfway’ running, most proteins remained stacked in a small area of approximately 2 mm in height between the gel boundary and the blue marker. After electrophoresis, this small gel area was excised from the gel slab, and the proteins were fixed in the gel slice with an excess volume of aqueous solution containing 40% methanol and 10% acetic acid.

### In-gel tryptic digestion of protein

The gel slice was subjected to an in-gel tryptic digestion process (Shevchenko *et al*, 1996), with minor modifications. Briefly, after *S*-carboxyamidomethylation of Cys residues with iodoacetamide, the gel slice was incubated in a small volume of 50 mM ammonium bicarbonate buffer solution containing 1 *μ*g of trypsin (Promega Co., Madison, WI, USA). The resulting peptides were extracted from the gel matrix, and dried under vacuum.

### Liquid chromatography-tandem mass spectrometry

The peptide mixture (1 *μ*g) was analysed using a liquid chromatography-tandem mass spectrometry (LC-MS/MS) system in a fully automated manner (Kawakami *et al*, 2005). Briefly, reversed-phase peptide separation was performed on a C18 capillary LC column (Michrom BioResources Inc., Auburn, CA, USA) at a flow rate of 1 *μ*l/min. For gasification of the protonated peptides, the LC effluent was directly interfaced with an electrospray ionisation (ESI) source in a positive ion mode modified on a Finnigan LTQ linear ion trap mass spectrometer (Thermo Fisher Scientific Inc., Waltham, MA, USA) (Schwartz *et al*, 2002). The ESI used a Teflon-coated spray needle (20 *μ*m id, AMR Inc., Tokyo, Japan). The ESI-MS/MS operation and continuous data acquisition of full MS scan and subsequent three MS/MS scans were carried out on an Xcalibur system controller (Thermo Fisher Scientific).

### Semiquantitative analysis

All full MS data were investigated using an i-OPAL semiquantitative LC-MS data analysis system (i-OPAL algorithm: Patent no. WO 2004/090526 AI). First, the signal intensity of the full MS scan was normalised so that the total signal intensity of each sample became the same value. Several standard signals derived either from the injected egg white lysozyme or from sample intrinsic common proteins were selected as i-OPAL alignment markers. The i-OPAL alignment programme was used to align the nonlinearly fluctuating LC retention time axis of all LC-MS data to finally generate a single combined LC-MS data set for the soluble and the insoluble fractions, respectively. Analysis of variance (ANOVA) was applied for each peak signal in the final combined LC-MS data set to select candidate marker signals whose intensity differed significantly in a particular patient group. ANOVA was carried out using a Spotfire DecisionSite package.

### Database searches

All MS/MS data were investigated using the Mascot search engine (Matrix Science Ltd., London, UK, http://www.matrixscience.com) against the *Homo sapiens* (human) subset of the Swiss-Prot and the RefSeq protein sequence databases. The database searches were performed allowing for fixed modification of cysteine residues (*S*-carbamidomethylation, +57.0 Da) and variable modification of methionine residues (oxidation, +16.0 Da), peptide mass tolerance ±2.0 Da and fragment *m/z* tolerance ±0.8.

### Surgical specimens of stage I lung adenocarcinoma for immunohistochemical staining

#### Sample set A for confirmation of LC-MS semiquantitative results

To confirm the semiquantitative results of LC-MS, 23 formalin-fixed, paraffin-embedded specimens derived from the same cases as those used for LC-MS analysis were collected for immunohistochemical investigation. As one formalin-fixed specimen had already been exhausted for the previous investigations, the remaining 23 specimens were investigated.

#### Sample set B for validation

To validate the expression of the protein molecules on lung adenocarcinoma cells, 90 formalin-fixed, paraffin-embedded specimens from patients with lung adenocarcinoma, resected at Tokyo Medical University Hospital between 1995 and 2001, were used. All the patients had undergone curative resection of lung cancer, and after surgery, a pathologically definitive diagnosis of stage I adenocarcinoma had been obtained. We evaluated recurrence after surgery using chest roentgenography and serum tumour markers (CEA, CA19-9 and SLX) every 3 months and computed tomography of the head and body, and bone scintigraphy, every 6 months. When it was difficult to evaluate roentgenographically whether the lesion was recurrent or not, either cytological or pathological examinations were performed to obtain a definitive diagnosis (Table 1).

Of the 90 patients, 51 underwent PAC using uracil–tegafur. These 51 cases included 24 recurrences (U1R1) within 5 years and 27 cases without recurrence (U1R0) within 5 years after surgical treatment. The remaining 39 patients did not receive any adjuvant chemotherapy. These 39 patients included 17 with recurrence (U0R1) and 22 without recurrence (U0R0). The clinicopathological backgrounds of the 90 patients with lung adenocarcinoma are summarised in Table 2.

### Immunohistochemical staining of surgically resected specimens of stage I lung adenocarcinoma

Four-micrometer-thick tissue sections were prepared from formalin-fixed, paraffin-embedded surgical specimens and collected on glass slides. The sections were stained immunohistochemically by the ABC method using either anti-myosin IIA mouse monoclonal antibody (clone ab24762, abcam, Cambridge, CB4 0FW, UK) (diluted 1 : 500) or anti-vimentin antibody (Dako Cytomation, Denmark A/S) (diluted 1 : 100) as the first antibody. After deparaffinisation, specimens were treated with 0.01% trypsin and an autoclave antigen retrieval system (Barbareschi *et al*, 1994). Sequentially, after inhibition of endogenous peroxidase activity with 0.5% hydrogen peroxide and incubation with 2% normal swine serum, the first antibody was applied. Biotinylated anti-mouse immunoglobulin (Vector Laboratories Inc., Burlingam, CA, USA) was applied as the second antibody (diluted 1 : 200), followed by application with avidin–biotin peroxidase complex (Vector Laboratories Inc.) (diluted 1 : 100). The specimens were reacted with 0.06% 3,3’-diaminobenzidine tetrahydrochloride and 0.03% hydrogen peroxide in Tris-buffered saline to visualise the positive areas. Meyer’s haematoxylin was used for counterstaining.

### Evaluation of immunohistochemically stained preparations

Cells showing cytoplasmic staining were evaluated as positive. For myosin IIA immunohistochemical staining, we evaluated a case as positive when more than 50% of the cells were stained. Also, for vimentin immunostaining, cases in which more than 25% of the cells were stained were evaluated as positive. For both kinds of staining, normal alveolar epithelium served as an internal negative control.

### Statistical analysis

Statistical analysis was carried out using the SPSS program. Statistical significance of the relationship between recurrence and immunohistochemical reactivity was evaluated using χ^2^ test. Disease-free survival curves were calculated from the day of surgery using the Kaplan–Meier method, and the significance of differences in survival rates between the patient groups was calculated by the log-rank test. In all statistical analyses, a *P*-value of <0.05 was taken to indicate a statistically significant difference.

## RESULTS

### LC-MS data analysis

After i-OPAL alignment and peak detection, we obtained 13 136 signal peaks from the soluble fraction and 14 984 peaks from the insoluble fraction. Using Spotfire, we restricted the candidate signal peaks on the basis of the following conditions:

1. A Mascot search result with a score equal to or more than 50.
2. An ANOVA *P*-value equal to or less than 1 × 10^−5^ (for the soluble fraction) or 1 × 10^−6^ (for the insoluble fraction).

**Figure 1:**
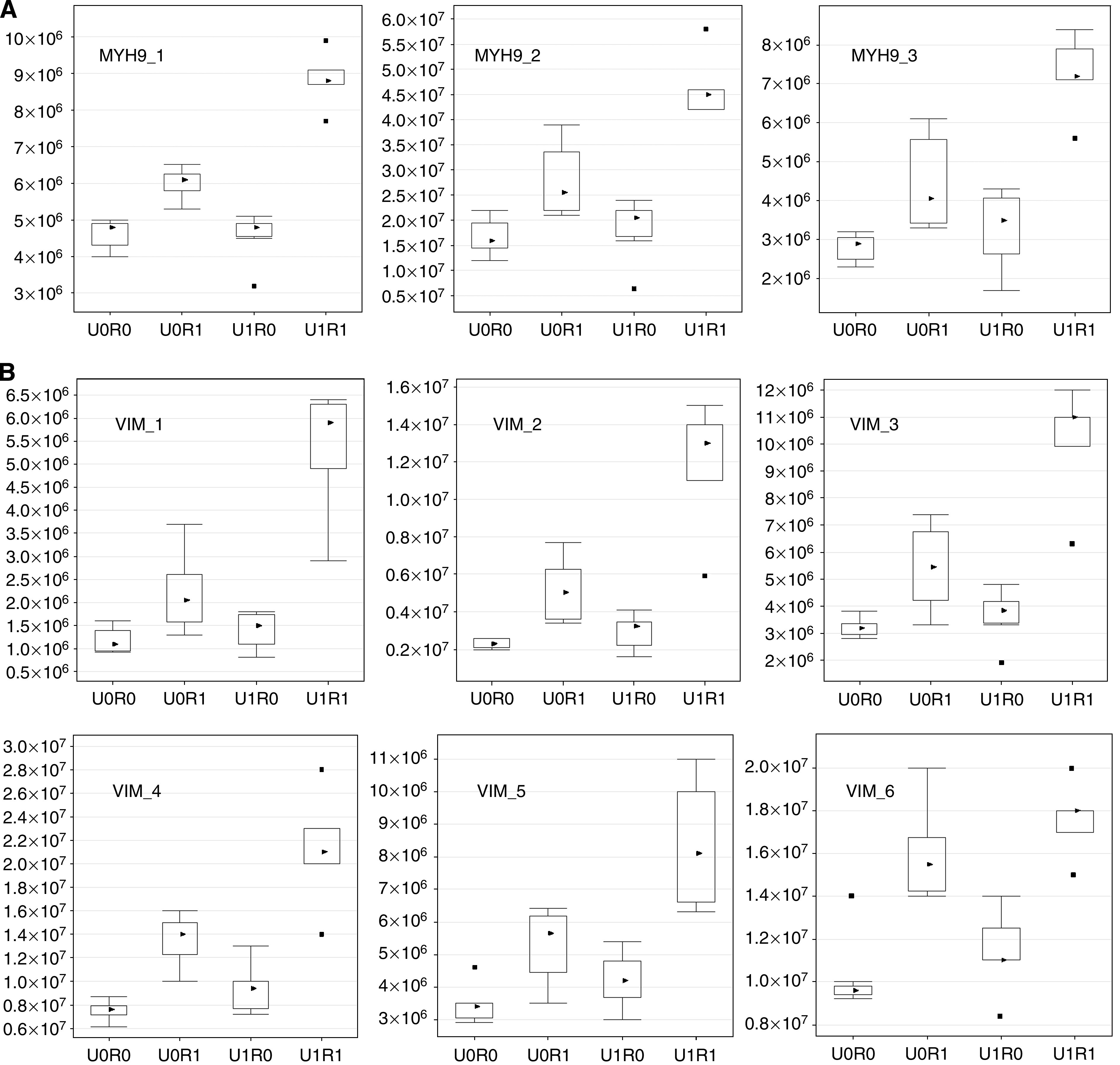
Comparison of the intensity of peptide signals originating from the same protein molecule in each group detected by LC-MS. The vertical axis indicates normalised signal intensity measured by LC-MS. In each box plot, the upper and lower sides of the box represent the upper and the lower quartile values (Q3/Q1), and the upper and lower horizontal bars outside the box indicate the upper and the lower adjacent values (UAV/LAV). Note that UAV is the largest observation value that is less than or equal to Q3+1.5 × (Q3–Q1), and LAV is the smallest observation greater than or equal to Q1–1.5 × (Q3–Q1). Black triangle marks represent the median values, and black square marks represent outliers. U0R0: patients without PAC showing no recurrence within 5 years after surgery. U0R1: patients without PAC in showing recurrence within 5 years after surgery. U1R0: patients who received PAC with uracil–tegafur and showed no recurrence within 5 years after surgery. U1R1: patients who received PAC with uracil–tegafur and showed recurrence within 5 years after surgery. (**A**) These three peptide signals were shown by MS/MS to have originated from myosin IIA. There was a significant difference between the U1R1 and the other groups (*P*<9.7 × 10^−7^). (**B**) These six peptide signals were shown by MS/MS to have originated from vimentin. There was also a significant difference between the U1R1 and the other groups (*P*<8.3 × 10^−6^).

As the peptide compositions of the soluble and the insoluble fractions differed, we applied different criteria to obtain approximately the same number of candidate signals. As a result, we were able to restrict the number of candidate signals to 23 and 28 for the soluble and insoluble fractions, respectively. From the restricted candidate signals, we selected several myosin IIA and vimentin signals as final candidate biomarker signals, because these two candidate biomarkers were identified by more than one distinct peptide sequence, and almost all of these signals had similar patterns of intensity (Figure 1A and B; Table 3).

Table 3 lists the amino-acid sequences from the selected candidate biomarker signals described above. These sequences were identified from MS/MS data using Mascot software.

Figure 1A shows the distribution of the signal intensity of several peptide ions derived from myosin IIA, and Figure 1B shows the signal intensity distribution of vimentin-derived peptide ions. For most signals, the intensity for group U1R1 patients showed patterns that differed significantly (i.e., were markedly higher) from those of the other patient groups.

### Immunohistochemical staining of myosin IIA and vimentin

**Figure 2:**
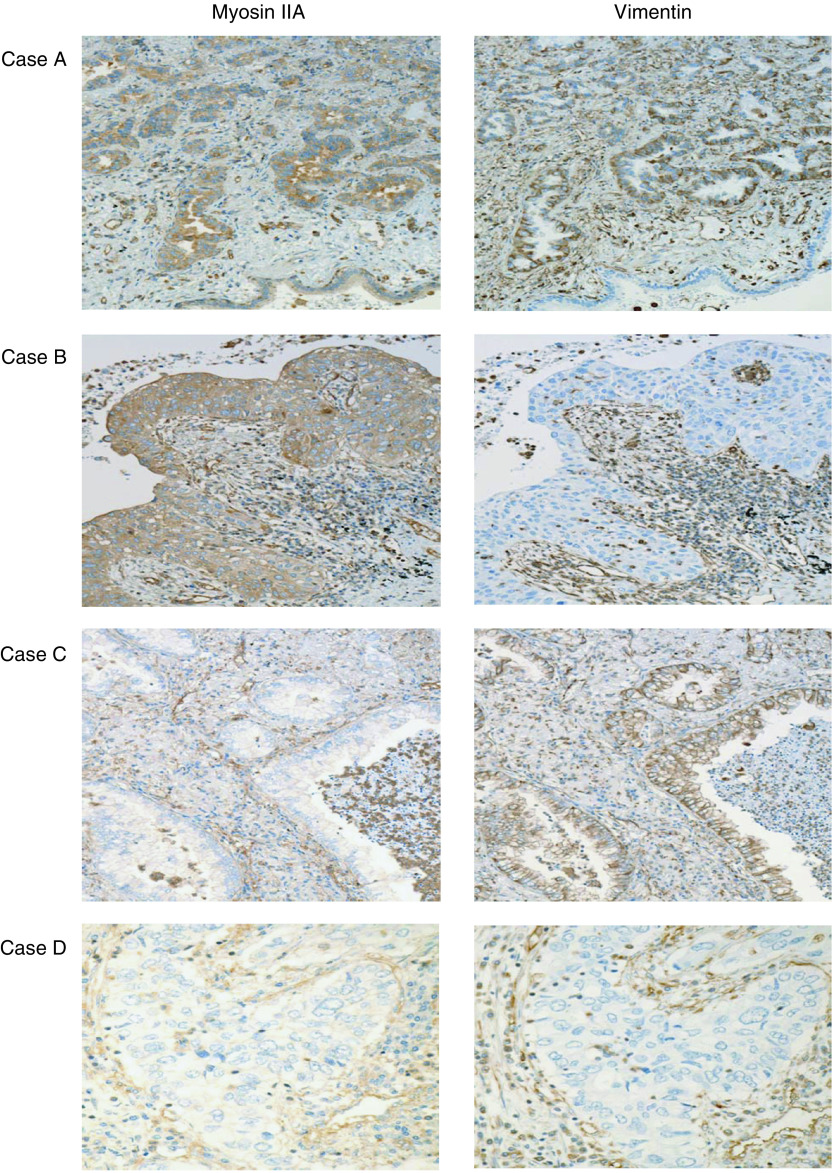
Immunohistochemical reactivity of representative cases using anti-myosin IIA antibody, ab24762 (abcam, Cambridge, UK) and anti-vimentin antibody (Dako Cytomation, Denmark A/S). Case A showed positive cytoplasmic staining for both myosin IIA and vimentin. Case B showed positive cytoplasmic staining for myosin IIA and negative cytoplasmic staining for vimentin. Case C showed negative cytoplasmic staining for myosin IIA and positive cytoplasmic staining for vimentin. Case D showed negative cytoplasmic staining for both myosin IIA and vimentin.

Representative staining of myosin IIA and vimentin is shown in Figure 2, and a summary of the immunohistochemical data is presented in Table 4. Cytoplasmic staining was observed in cases positive for myosin IIA and vimentin. We evaluated cases in which more than 50% of the cells showed immunohistochemical reactivity for myosin IIA, considered as overexpressing (positive). We also evaluated overexpressing (positive) cases in which more than 25% of the cells showed immunohistochemical reactivity for vimentin. On the basis of these criteria, we evaluated sample sets A and B.

Immunohistochemical evaluation of sample set A (Table 4A): all patients with cancers lacking expression of both myosin IIA and vimentin showed relapse-free survival at 5 years. On the other hand, all patients with cancers showing positive expression of both myosin IIA and vimentin suffered disease recurrence.

Immunohistochemical evaluation of sample set B (Table 4B): among 90 cases, 75 (83.3%) showed overexpression of myosin IIA, and 48 (53.3%) showed overexpression of vimentin. There was no relationship between the immunohistochemical reactivities of myosin IIA and vimentin. All nine patients whose cancers lacked immunohistochemical reactivity for both myosin IIA and vimentin showed relapse-free survival at 5 years. Among cases that were immunohistochemically negative for both myosin IIA and vimentin, we recognised a statistically significant difference between U1R1 and U0R0 (*P*=0.008), but there were no significant differences between U1R1 and the other groups.

### Disease-free survival and coexpression of myosin IIA and vimentin in sample set B

**Figure 3:**
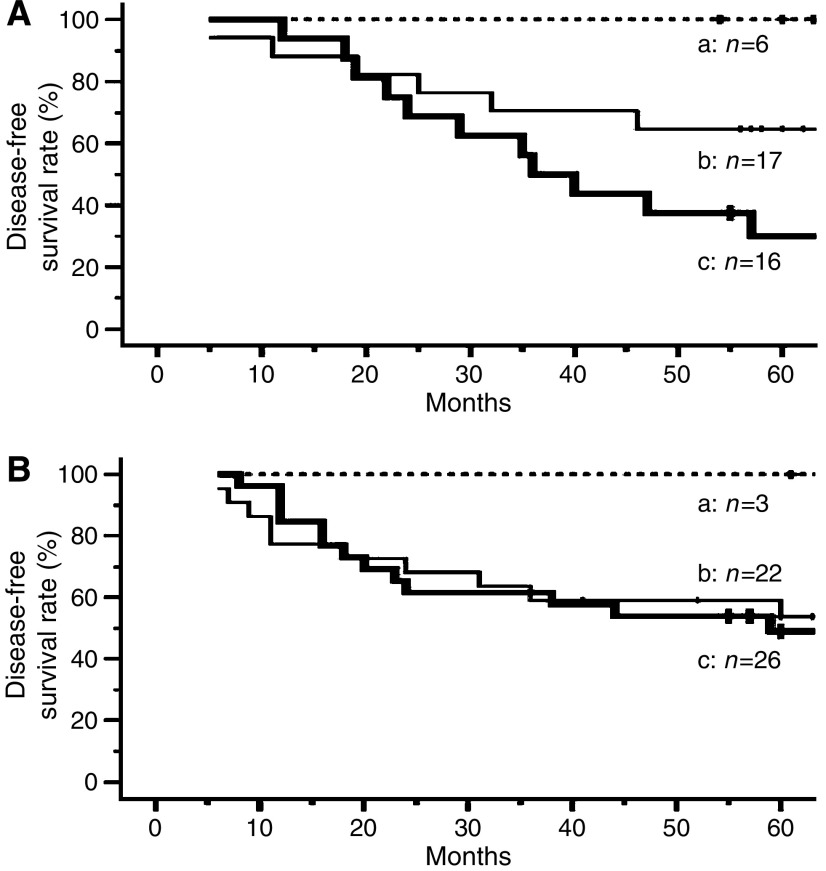
Kaplan–Meier curves for disease-free survival after complete resection in patients with stage I lung adenocarcinoma who received PAC with uracil–tegafur (**A**), or did not receive any PAC (**B**). a: Cases lacking both myosin IIA and vimentin expression (non-relapse survival rate at 5 years: 100% in panels **A** and **B**). b: Cases negative for myosin IIA expression and positive for vimentin expression, or positive for myosin IIA and negative for vimentin expression (non-relapse survival rate at 5 years: 64.7% in panel **A** and 53.7% in panel **B**). c: Cases positive for both myosin IIA and vimentin expression (non-relapse survival rate at 5 years: 30.0% in panel **A** and 49.0% in panel **B**). In patients who did not receive adjuvant chemotherapy, there was a statistically significant difference in disease-free survival between those who were negative and those who were positive for both proteins (a–c: *P*=0.011). No significant difference in this respect was recognised in patients who received PAC with uracil–tegafur.

The non-relapse survival curves of cases with/without PAC are shown in Figure 3A and B. Irrespective of whether patients had undergone PAC or not, the non-relapse survival rate of cases lacking expression of both myosin IIA and vimentin was 100%. Among patients who had not undergone PAC, there was a statistically significant difference between cases lacking expression of both myosin IIA and vimentin and cases that were positive for both (*P*=*0.011*) (Figure 3A). Among the patients who received PAC, there was no statistically significant difference in this respect (Figure 3B). When the cases showing positive expression of both myosin IIA and vimentin were evaluated, we recognised a 5-year-survival rate benefit of approximately 19% in patients who had undergone PAC with uracil–tegafur, but there was no statistically significant difference in this respect between patients who had and who had not received PAC.

**Figure 4:**
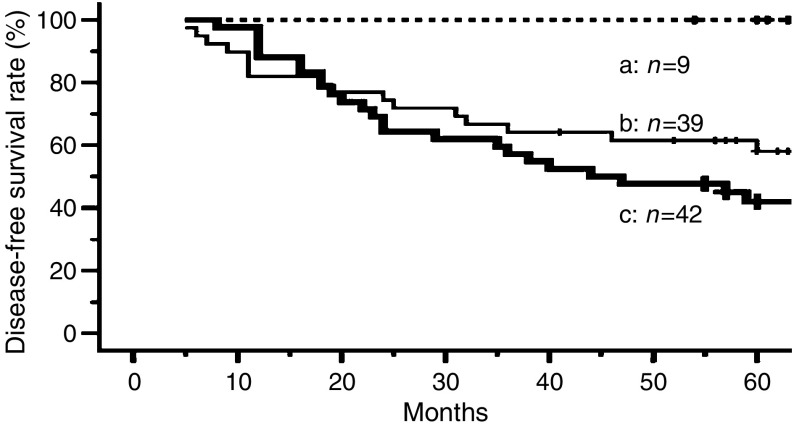
Kaplan–Meier curves for disease-free survival after complete resection in patients with stage I lung adenocarcinoma. a: Cases lacking both myosin IIA and vimentin expression (non-relapse survival rate at 5 years: 100%). b: Cases negative for myosin IIA and positive for vimentin, or positive for myosin IIA and negative for vimentin (non-relapse survival rate at 5 years: 58.0%). c: Cases positive for both myosin IIA and vimentin (non-relapse survival rate at 5 years: 42.0%). Group a: showed significantly higher survival than group b, and significantly higher survival than group c: (a–b: *P*=0.029; a–c: *P*=0.006).

When we evaluated the non-relapse survival curves of all the studied cases, there were statistically significant differences between cases negative for myosin IIA and vimentin expression and cases that were positive for both (*P*=*0.006*), and between cases positive for either myosin IIA or vimentin and cases that were negative for both (*P*=*0.029*; Figure 4).

## DISCUSSION

Lung cancer is the leading cause of cancer death in Japan, and its incidence is still increasing. Even if surgical resection involving either lobectomy or pneumonectomy accompanied by lymph node dissection is performed at a relatively early stage, distant metastasis often occurs within a few years. More than 20% of patients with stage I NSCLC suffer recurrence caused by distant metastasis. Distant metastasis is the most frequent mode of recurrence in patients who undergo surgical resection of lung cancer, and it is believed that in such patients, micrometastasis is invariably present at the time of initial treatment. If an efficient PAC regimen could be devised for total control of micrometastasis, then the prognosis of patients with lung cancer would be markedly improved. A meta-analysis conducted in the 1990s showed that PAC using platinum-based agents had no effect on the survival of patients with NSCLC, even though previous studies had suggested a 5% increase in survival at 5 years (Non-Small Cell Lung Cancer Collaborative Group, 1995). At the 2004 ASCO meeting, the results of two randomised adjuvant trials showing the efficacy of platinum-based chemotherapy – the CALGB-9633 trial (carboplatin and paclitaxcel) (Strauss *et al*, 2004) and the JBR 10 trial (cisplatin and vinorelbine) – were reported. Furthermore, a recent large-scale randomised clinical trial involving meta-analysis concluded that patients assigned to cisplatin-based PAC had a significantly higher survival rate than those assigned to postoperative observation (44.5 *vs* 40.4% at 5 years; *P*<0.03) (Arriagada *et al*, 2004). Also our previous study showed that PAC with uracil–tegafur conferred a survival benefit for patients with resected stage I adenocarcinoma of the lung (Kato *et al*, 2004). Also, meta-analysis of PAC with tegafur–uracil supported this result (Hamada *et al*, 2005). However, even though a significant difference was found in this study, the 5-year-survival rate benefit of this therapy was 11.5% for stage IB adenocarcinoma (Kato *et al*, 2004). At present, although leading lung cancer experts appear to have reached a consensus concerning the effectiveness of PAC, none of the present PAC regimens are of benefit to more than 15% of patients with NSCLC. In this context, it is very important to predict the response to PAC and to select potential responders before carrying out PAC. Therefore, we attempted to identify biomarkers of either responders or nonresponders including relapse-free patients without PAC using uracil–tegafur, for selection of patients who would benefit from PAC using proteomic analysis of surgically resected specimens of stage I lung adenocarcinoma.

As proteins play a role in both physiological and pathological functions, it is now recognised that investigation of proteins is essential to obtain an accurate grasp of cellular physiology. Recent advances in proteomic techniques, including two-dimensional polyacrylamide gel electrophoresis and MS, have brought hope that the pathogenesis of any type of malignant neoplasm will be ultimately clarified. We believe that the present concepts of proteomic analysis will prove to be extremely valuable in the field of clinical oncology, and will lead to the development of new therapeutic strategies. Liquid chromatography-tandem mass spectrometry enables simultaneous evaluation of a large number of polypeptides, and furthermore, MS/MS has made it possible to identify protein molecules by obtaining information about their amino-acid sequences. We attempted to identify proteins associated with the effectiveness of postoperative uracil–tegafur chemotherapy and the favourable prognosis of stage I adenocarcinoma, and detected two kinds of protein molecules (myosin IIA and vimentin) showing significantly high expression in the group that suffered recurrence despite administration of uracil–tegafur, in comparison with the other groups. Our semiquantitative results of LC-MS were confirmed by immunohistochemistry for myosin IIA and vimentin (Table 4A).

Nonmuscle myosin IIA is a major component of the actomyosin cytoskeleton and is generally considered to contribute to contraction of the cell posterior during migration (Ridley *et al*, 2003). However, there is still a profound lack of understanding of the exact mechanical roles of myosin IIA during cell migration. A recent clinical study of patients with NSCLC found a significant positive correlation between the expression levels of myosin light chain kinase (which activates myosin II) and the likelihood of disease recurrence and metastasis (Minamiya *et al*, 2005), indicating that myosin IIA activation could be a factor contributing to metastasis. A key role for myosin IIA in cancer cell metastasis has been further suggested, indirectly, by a number of published studies focusing on the small calcium-binding protein, metastasin-1. This protein is upregulated in many metastasis cell lines, and when overexpressed enhances metastatic behaviour (Davies *et al*, 1993). A major cellular target of metastasin-1 seems to be myosin IIA (Garrett *et al*, 2006). Although studies of metastasin-1 suggest critical roles for myosin IIA in metastasis, it remains completely unknown how myosin IIA contributes to metastasis, and which isoforms are important for this process.

Vimentin is the most ubiquitous intermediate filament protein and the first to be expressed during cell differentiation. All primitive cell types express vimentin, but in most nonmesenchymal cells, it is replaced by other intermediate filament proteins during differentiation. Vimentin is expressed in a wide variety of mesenchymal cell types (fibroblasts, endothelial cells, etc), and also in a number of other cell types derived from mesoderm, mesothelium and ovarian granulose cells. Epithelial–mesenchymal transition is a key mechanism operating in the normal development of multicellular organisms. During this process, epithelial cells progressively acquire a reversible or irreversible mesenchymal phenotype that is essential for organogenesis (Thiery, 2002). Morphogenetic epithelial–mesenchymal transition is aberrantly recapitulated during tumorigenesis in a variety of epithelial cancers, including those of the thyroid, liver, kidney, prostate, breast and lung (Arias, 2001; Thiery, 2002). The common signature of this process involves disruption of normal epithelial integrity, with loss of morphological features including polarised epithelia, and partial or total gain of mesenchymal markers with progressive acquisition of a motile and invasive phenotype (Islam *et al*, 1996). In addition to a disrupted epithelial morphology, dysregulation of adhesion and junctional molecules and aberrant expression of *N*-cadherin, epithelial–mesenchymal transition involves *de novo* expression of other mesenchymal markers, such as fibronectin and vimentin in epithelial cells. Aberrant expression of vimentin in tumours and transformed cell lines has been correlated with increased motility, invasive behaviour and poor prognosis (Gilles *et al*, 1996; Hendrix *et al*, 1997). Recently, it was reported that the presence of vimentin-positive tumour cells mainly in fibrotic areas is consistent with other studies that have shown a correlation between tumour fibrosis and epithelial–mesenchymal transition (Blanco *et al*, 2004).

These two molecules identified by proteomic analysis might reflect the cellular functions of metastasis and the mechanism of recurrence of malignant neoplasms. We attempted to validate the results of LC-MS/MS using immunohistochemistry of an additional sample set (sample set B: 90 surgically resected lung cancer specimens) with monoclonal antibodies against the two proteins. The results showed that cases lacking expression of the two proteins had a good prognosis, irrespective of whether the patients had undergone PAC. Therefore, these two proteins appear to be potentially useful biomarkers for the selection of patients who do not require PAC. In the cases positive for both of these proteins, the 5-year-survival benefit was approximately 19% in patients with adenocarcinoma who underwent PAC with uracil–tegafur. However, there was no significant difference between patients who did, and did not, undergo PAC with uracil–tegafur. Therefore, in this investigation, we failed to select patients who might benefit from this adjuvant chemotherapy. A larger-scale investigation is therefore needed to establish suitable biomarkers for the selection of patients who might benefit from PAC with uracil–tegafur, because a few per cent of patients with stage I adenocarcinoma do obtain such a benefit.

Individualised chemotherapy for lung cancer patients is currently attracting attention, because the efficacy of systemic chemotherapy using any single agent is less than 30%. Therefore, it is extremely important to select patients who might benefit from chemotherapy. Until an ideal chemotherapy agent is established, we propose that rather than focusing only on improving the efficacy of chemotherapy regimens, we should also make efforts to identify patients who will show a good response to regimens that are already established. This proposal is justified only on the basis of evidence-based medicine. In this situation, positive indicators for the effectiveness of PAC with uracil–tegafur are needed. However, in this study, we were unable to detect novel biomarkers for selection of good responders. The two protein molecules detected in this proteomic analysis were biomarkers indicative of good prognosis.

The ultimate purpose of clinical proteomics is to improve diagnostic procedures including the exact evaluation of biological characteristics of tumour cells and to understand the molecular pathogenesis of cancers to devise novel therapeutic strategies. We believe that proteomic analysis will become an integral tool for investigation of tumour biology. We conclude that negative expression of both myosin IIA and vimentin is an indicator of good prognosis for stage I lung adenocarcinoma without the need for PAC.

## Figures and Tables

**Table 1:** Clinical features of lung adenocarcinoma cases subjected to LC-MS/MS

**Characteristic**	**(%)**
*Age (year)*	
Median	65.0
Range	32–78
	
*Gender*	
Male	19 cases (79.2%)
Female	5 cases (20.8%)
	
*Pathological stage*	
IA	10 cases (41.7%)
IB	14 cases (58.3%)
	
*Presence of recurrence*	
(+)	11 cases (45.8%)
(−)	13 cases (54.2%)
	
*PAC*	
(+)	11 cases (45.8%)
(−)	13 cases (54.2%)

PAC=postoperative adjuvant chemotherapy with oral uracil–tegafur.

**Table 2:** Clinical features of lung adenocarcinoma cases as revealed by immunohistochemical staining

**Characteristic**	**(%)**
*Age (year)*	
Median	64.8
Range	45–82
	
*Gender*	
Male	54 cases (60.0%)
Female	36 cases (40.0%)
	
*Pathological stage*	
IA	33 cases (36.7%)
IB	57 cases (63.3%)
	
*Existence of recurrence*	
(+)	41 cases (45.6%)
(−)	49 cases (54.4%)
	
*PAC with urasil-tegafur*	
(+)	51 cases (56.7%)
(−)	39 cases (43.3%)

PAC=postoperative adjuvant chemotherapy.

**Table 3:** Amino-acid sequences from the selected peptide ion signals

**Name**	**Fraction**	**Sequence**
*Myosin, heavy polypeptide 9, non-muscle*		
MYH9_1	Insoluble	IRELESQISELQEDLESER
MYH9_2	Insoluble	KANLQIDQINTDLNLER
MYH9_3	Insoluble	HEMPPHIYAITDTAYR
		
*Vimentin*		
VIM_1	Insoluble	ETNLDSLPLVDTHSK
VIM_2	Insoluble	NLQEAEEWYK
VIM_3	Insoluble	LGDLYEEEMR
VIM_4	Insoluble	LLQDSVDFSLADAINTEFK
VIM_5	Soluble	SGDAAIVDMVPGKPMCVESFSDYPPLGR
VIM_6	Soluble	ILTVEDHYYEGGIGEAVSSAVVGEPGITVTHLAVNR

**Table 4:** Relationship between PAC, recurrence and immunohistochemical reactivity for myosin IIA and vimentin

	**M(−)V(−)**	**M(−)V(+) or M(+)V(−)**	**M(+)V(+)**	**ND**
*(A) Sample set A (n=24) derived from the same cases as those subjected to LC-MS/MS*				
U0R0	5	2	0	0
U1R0	2	3	0	1
U0R1	0	2	4	0
U1R1	0	0	5	0
				
*(B) Sample set B (n=90) for validation by immunohistochemical analysis*				
U0R0	6	11	5	0
U1R0	3	12	12	0^*^
U0R1	0	6	11	0
U1R1	0	10	14	0

M=expression of myosin IIA; ND=not done; U0R0=patients without PAC showing no recurrence within 5 years after surgery; U0R1=patients without PAC showing recurrence within 5 years after surgery; U1R0=patients who received PAC with uracil–tegafur and showed no recurrence within 5 years after surgery; U1R1=patients who received PAC with uracil–tegafur and showed recurrence within 5 years after surgery; V=expression of vimentin.

^*^Statistically significant difference between U0R0 and U1R1 was detected (*P*=0.008).
