# Proteomic analysis of stage I primary lung adenocarcinoma aimed at individualization of postoperative therapy

**DOI:** 10.1038/sj.bjc.6604414

**Published:** 2008-06-10

**Authors:** J Maeda, T Hirano, A Ogiwara, S Akimoto, T Kawakami, Y Fukui, T Oka, Y Gong, R Guo, H Inada, K Nawa, M Kojika, Y Suga, T Ohira, K Mukai, H Kato

**Correction to**: *British Journal of Cancer* (2008) **98**, 596–603; doi:10.1038/sj.bjc.6604197

Owing to an error on the part of the authors, mistakes were made in the assignment of protein names to the peptides found as biomarkers in the above paper.

The last two graphs in Figure 1 entitled ‘VIM_5’ (lower middle) and ‘VIM_6’ (lower right) should be removed. They are not vimentin-derived peptide signals. The caption of Figure 1B should also be corrected from ‘These six peptide signals’ to ‘These four peptide signals’. Also in the last part of the caption, ‘*P*<8.3 × 10^−6^’ should be changed as ‘*P*<3.8 × 10^−7^’.

In the legend to Figure 3, an error was made in the description of the two graphs (A and B), with the graphs being transposed. The first line of the legend should have read: ‘Kaplan–Meier curves for disease-free survival after complete resection in patients with stage I lung adenocarcinoma who received PAC with uracil-tegafur (B), or did not receive any PAC (A).’

The last two rows of Table 3 should also be removed. The amino-acid sequences assigned as ‘VIM_5’ and ‘VIM_6’ should not be assigned as Vimentin.

## Figures and Tables

**Figure 1:**
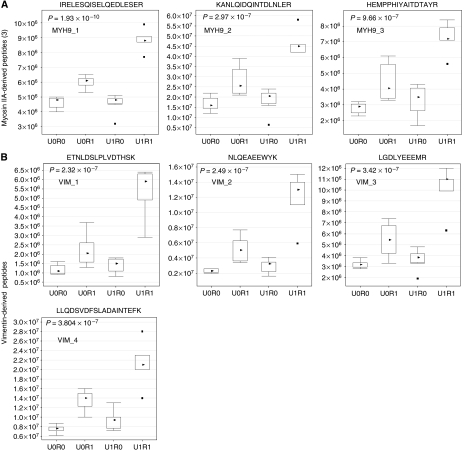
Comparison of the intensity of peptide signals originating from the same protein molecule in each group detected by LC-MS. The vertical axis indicates normalised signal intensity measured by LC-MS. In each box plot, the upper and lower sides of the box represent the upper and the lower quartile values (Q3/Q1), and the upper and lower horizontal bars outside the box indicate the upper and the lower adjacent values (UAV/LAV). Note that UAV is the largest observation value that is less than or equal to Q3+1.5 × (Q3–Q1), and LAV is the smallest observation greater than or equal to Q1–1.5 × (Q3–Q1). Black triangle marks represent the median values, and black square marks represent outliers. U0R0: patients without PAC showing no recurrence within 5 years after surgery. U0R1: patients without PAC showing recurrence within 5 years after surgery. U1R0: patients who received PAC with uracil-tegafur and showed no recurrence within 5 years after surgery. U1R1: patients who received PAC with uracil-tegafur and showed recurrence within 5 years after surgery. (**A**) These three peptide signals were shown by MS/MS to have originated from myosin IIA. There was a significant difference between the U1R1 and the other groups (*P*<9.7 × 10^−7^). (**B**) These four peptide signals were shown by MS/MS to have originated from vimentin. There was also a significant difference between the U1R1 and the other groups (*P*<3.8 × 10^−7^).

**Table 3:** Amino-acid sequences from the selected peptide ion signals

**Name**	**Fraction**	**Sequence**
*Myosin, heavy polypeptide 9, non-muscle*		
MYH9_1	Insoluble	IRELESQISELQEDLESER
MYH9_2	Insoluble	KANLQIDQINTDLNLER
MYH9_3	Insoluble	HEMPPHIYAITDTAYR
		
*Vimentin*		
VIM_1	Insoluble	ETNLDSLPLVDTHSK
VIM_2	Insoluble	NLQEAEEWYK
VIM_3	Insoluble	LGDLYEEEMR
VIM_4	Insoluble	LLQDSVDFSLADAINTEFK

